# 2,4-Dichloro-*N*-(2,5-dimethyl­phen­yl)benzene­sulfonamide

**DOI:** 10.1107/S1600536811036968

**Published:** 2011-09-17

**Authors:** Vinola Z. Rodrigues, Sabine Foro, B. Thimme Gowda

**Affiliations:** a Department of Chemistry, Mangalore University, Mangalagangotri 574 199, Mangalore, India; b Institute of Materials Science, Darmstadt University of Technology, Petersenstrasse 23, D-64287 Darmstadt, Germany

## Abstract

The asymmetric unit of the title compound, C_14_H_13_Cl_2_NO_2_S, contains three independent moleules. The torsion angles of the C—SO2—NH—C segments in the three mol­ecules are 67.5 (2), 83.4 (2) and −77.5 (2)°. The two aromatic rings are tilted relative to each other by 68.8 (1), 64.1 (1) and 68.5 (1)°. The crystal structure features dimers linked by pairs of N—H⋯O hydrogen bonds.

## Related literature

For the preparation of the title compound, see: Savitha & Gowda (2006 ▶). For hydrogen-bonding modes of sulfonamides, see: Adsmond & Grant (2001 ▶). For our studies on the effects of substituents on the structures and other aspects of *N*-(ar­yl)-amides, see: Arjunan *et al.* (2004 ▶); Gowda *et al.* (2000 ▶), on *N*-(ar­yl)-methane­sulfonamides, see: Gowda *et al.* (2007 ▶) and on *N*-(ar­yl)-aryl­sulfonamides, see: Gelbrich *et al.* (2007 ▶); Perlovich *et al.* (2006 ▶); Gowda *et al.* (2010 ▶).
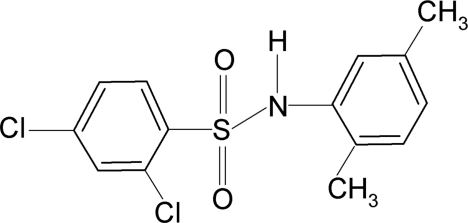


## Experimental

### 

#### Crystal data

C_14_H_13_Cl_2_NO_2_S
*M* *_r_* = 330.21
Monoclinic, 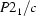

*a* = 24.3070 (8) Å
*b* = 14.8880 (6) Å
*c* = 12.4365 (5) Å
β = 94.929 (3)°
*V* = 4483.9 (3) Å^3^
*Z* = 12
Mo *K*α radiation
μ = 0.57 mm^−1^
*T* = 293 K
0.42 × 0.36 × 0.36 mm

#### Data collection

Oxford Diffraction Xcalibur diffractometer with a Sapphire CCD detector
Absorption correction: multi-scan (*CrysAlis RED*; Oxford Diffraction, 2009 ▶) *T*_min_ = 0.795, *T*_max_ = 0.820
18567 measured reflections
9151 independent reflections
6822 reflections with *I* > 2σ(*I*)
*R*_int_ = 0.015

#### Refinement

*R*[*F* ^2^ > 2σ(*F* ^2^)] = 0.038
*wR*(*F* ^2^) = 0.099
*S* = 1.01
9151 reflections
556 parameters
3 restraints
H atoms treated by a mixture of independent and constrained refinement
Δρ_max_ = 0.34 e Å^−3^
Δρ_min_ = −0.34 e Å^−3^

### 

Data collection: *CrysAlis CCD* (Oxford Diffraction, 2009 ▶); cell refinement: *CrysAlis RED* (Oxford Diffraction, 2009 ▶); data reduction: *CrysAlis RED*; program(s) used to solve structure: *SHELXS97* (Sheldrick, 2008 ▶); program(s) used to refine structure: *SHELXL97* (Sheldrick, 2008 ▶); molecular graphics: *PLATON* (Spek, 2009 ▶); software used to prepare material for publication: *SHELXL97*.

## Supplementary Material

Crystal structure: contains datablock(s) I, global. DOI: 10.1107/S1600536811036968/bt5642sup1.cif

Structure factors: contains datablock(s) I. DOI: 10.1107/S1600536811036968/bt5642Isup2.hkl

Supplementary material file. DOI: 10.1107/S1600536811036968/bt5642Isup3.cml

Additional supplementary materials:  crystallographic information; 3D view; checkCIF report

## Figures and Tables

**Table 1:** Hydrogen-bond geometry (Å, °)

*D*—H⋯*A*	*D*—H	H⋯*A*	*D*⋯*A*	*D*—H⋯*A*
N1—H1*N*⋯O5^i^	0.83 (2)	2.24 (2)	3.033 (2)	160 (2)
N2—H2*N*⋯O4^ii^	0.82 (2)	2.16 (2)	2.956 (2)	167 (2)
N3—H3*N*⋯O2^iii^	0.82 (2)	2.34 (2)	3.082 (2)	151 (2)

Symmetry codes: (i) 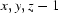; (ii) 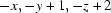; (iii) 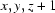.

**Fig. 1.**
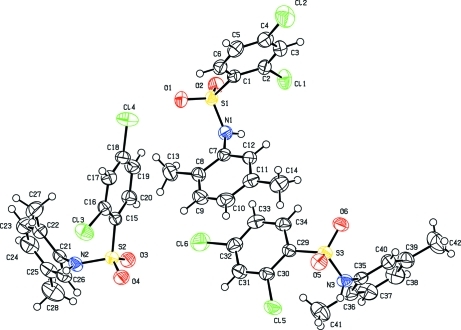
Molecular structure of the title compound, showing the atom labelling scheme and displacement ellipsoids are drawn at the 50% probability level.

**Fig. 2.**
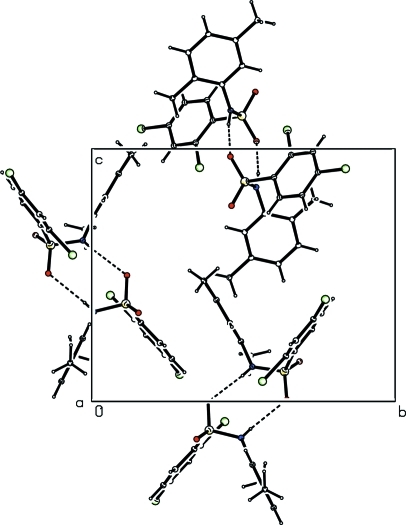
Molecular packing of the title compound with hydrogen bonding shown as dashed lines.

## supplementary crystallographic information

### Comment

The sulfonamide moiety is the constituent of many biologically important compounds. The hydrogen bonding preferences of sulfonamides have been investigated (Adsmond & Grant, 2001). As part of our studies on the substituent effects on the structures and other aspects of *N*-(aryl)-amides (Arjunan *et al.*, 2004; Gowda *et al.*, 2000), *N*-(aryl)-methanesulfonamides (Gowda *et al.*, 2007) and *N*-(aryl)-arylsulfonamides (Gowda *et al.*, 2010), in the present work, the crystal structure of 2,4-dichloro-*N*- (2,5-dimethylphenyl)-benzenesulfonamide (I) has been determined (Fig. 1).

The asymmetric unit of (I) contains three independent moleules. The molecules are twisted at the S atom with the C—SO_2_—NH—C torsion angles of 67.5 (2)° (molecule 1), 83.4 (2)° (molecule 2) and -77.5 (2)° (molecule 3), compared to the values of -85.1 (3)° and -47.2 (5)° in the major and minor components, respectively, of 2,4-dichloro-*N*-(2-methylphenyl)-benzenesulfonamide (II) (Gowda *et al.*, 2010)

The sulfonyl and the aniline benzene rings in (I) are tilted relative to each other by 68.8 (1)° in molecule 1, 64.1 (1)° in molecule 2 and 68.5 (1)° in molecule 3, compared to the values of 74.9 (1)° and 71.0 (3)° in the two components of(II)

The other bond parameters in (I) are similar to those observed in (II) and other aryl sulfonamides (Perlovich *et al.*, 2006; Gelbrich *et al.*, 2007).

In the crystal structure, the pairs of intermolecular N–H···O hydrogen bonds (Table 1) link the molecules into   dimers. Part of the crystal structure is shown in Fig. 2.

### Experimental

The solution of 1,3-dichlorobenzene (10 ml) in chloroform (40 ml) was treated dropwise with chlorosulfonic acid (25 ml) at 0 ° C. After the initial evolution of hydrogen chloride subsided, the reaction mixture was brought to room temperature and poured into crushed ice in a beaker. The chloroform layer was separated, washed with cold water and allowed to evaporate slowly. The residual 2,4-dichlorobenzenesulfonylchloride was treated with 2,5-dimethylaniline in the stoichiometric ratio and boiled for ten minutes. The reaction mixture was then cooled to room temperature and added to ice cold water (100 ml). The resultant solid 2,4-dichloro-*N*- (2,5-dimethylphenyl)-benzenesulfonamide was filtered under suction and washed thoroughly with cold water. It was then recrystallized to constant melting point from dilute ethanol. The purity of the compound was checked and characterized by recording its infrared and NMR spectra (Savitha & Gowda, 2006).

Prism like light pink single crystals used in X-ray diffraction studies were grown in ethanolic solution by slow evaporation at room temperature.

### Refinement

The H atoms of the NH groups were located in a difference map and refined with the N—H distance restrained to  0.86 (2) %A. The other H atoms were positioned with idealized geometry using a riding model with the aromatic C—H = 0.93Å and methyl C—H = 0.96 Å. Their isotropic displacement parameters  were set to 1.2*U*_eq_(C-aromatic, N) and 1.5*U*_eq_(C-methyl).

### Crystal data

**Table:**

C_14_H_13_Cl_2_NO_2_S	*F*(000) = 2040
*M**_r_* = 330.21	*D*_x_ = 1.467 Mg m^−^^3^
Monoclinic, *P*2_1_/*c*	Mo *K*α radiation, λ = 0.71073 Å
Hall symbol: -P 2ybc	Cell parameters from 7968 reflections
*a* = 24.3070 (8) Å	θ = 2.6–27.8°
*b* = 14.8880 (6) Å	µ = 0.57 mm^−^^1^
*c* = 12.4365 (5) Å	*T* = 293 K
β = 94.929 (3)°	Prism, light pink
*V* = 4483.9 (3) Å^3^	0.42 × 0.36 × 0.36 mm
*Z* = 12	

### Data collection

**Table:**

Oxford Diffraction Xcalibur diffractometer with a Sapphire CCD detector	9151 independent reflections
Radiation source: fine-focus sealed tube	6822 reflections with *I* > 2σ(*I*)
graphite	*R*_int_ = 0.015
Rotation method data acquisition using ω scans	θ_max_ = 26.4°, θ_min_ = 2.6°
Absorption correction: multi-scan (*CrysAlis RED*; Oxford Diffraction, 2009)	*h* = −26→30
*T*_min_ = 0.795, *T*_max_ = 0.820	*k* = −14→18
18567 measured reflections	*l* = −15→15

### Refinement

**Table:**

Refinement on *F*^2^	Primary atom site location: structure-invariant direct methods
Least-squares matrix: full	Secondary atom site location: difference Fourier map
*R*[*F*^2^ > 2σ(*F*^2^)] = 0.038	Hydrogen site location: inferred from neighbouring sites
*wR*(*F*^2^) = 0.099	H atoms treated by a mixture of independent and constrained refinement
*S* = 1.01	*w* = 1/[σ^2^(*F*_o_^2^) + (0.0428*P*)^2^ + 2.5197*P*] where *P* = (*F*_o_^2^ + 2*F*_c_^2^)/3
9151 reflections	(Δ/σ)_max_ = 0.001
556 parameters	Δρ_max_ = 0.34 e Å^−^^3^
3 restraints	Δρ_min_ = −0.34 e Å^−^^3^

### Special details

**Table:**

Geometry. All e.s.d.'s (except the e.s.d. in the dihedral angle between two l.s. planes) are estimated using the full covariance matrix. The cell e.s.d.'s are taken into account individually in the estimation of e.s.d.'s in distances, angles and torsion angles; correlations between e.s.d.'s in cell parameters are only used when they are defined by crystal symmetry. An approximate (isotropic) treatment of cell e.s.d.'s is used for estimating e.s.d.'s involving l.s. planes.
Refinement. Refinement of *F*^2^ against ALL reflections. The weighted *R*-factor *wR* and goodness of fit *S* are based on *F*^2^, conventional *R*-factors *R* are based on *F*, with *F* set to zero for negative *F*^2^. The threshold expression of *F*^2^ > σ(*F*^2^) is used only for calculating *R*-factors(gt) *etc*. and is not relevant to the choice of reflections for refinement. *R*-factors based on *F*^2^ are statistically about twice as large as those based on *F*, and *R*- factors based on ALL data will be even larger.

### Fractional atomic coordinates and isotropic or equivalent isotropic displacement parameters (Å^2^)

**Table:**

	*x*	*y*	*z*	*U*_iso_*/*U*_eq_	
C1	0.37117 (9)	0.66784 (14)	0.19972 (17)	0.0352 (5)	
C2	0.42435 (9)	0.64017 (15)	0.17972 (18)	0.0393 (5)	
C3	0.47007 (10)	0.67249 (17)	0.2413 (2)	0.0478 (6)	
H3	0.5054	0.6555	0.2264	0.057*	
C4	0.46238 (11)	0.73046 (17)	0.3255 (2)	0.0488 (6)	
C5	0.41066 (11)	0.75673 (16)	0.3491 (2)	0.0481 (6)	
H5	0.4063	0.7950	0.4068	0.058*	
C6	0.36519 (10)	0.72543 (15)	0.28586 (18)	0.0427 (5)	
H6	0.3300	0.7432	0.3012	0.051*	
C7	0.28443 (9)	0.49200 (14)	0.23739 (17)	0.0347 (5)	
C8	0.22861 (9)	0.48517 (16)	0.25563 (19)	0.0423 (5)	
C9	0.21695 (11)	0.43970 (17)	0.3489 (2)	0.0525 (6)	
H9	0.1803	0.4336	0.3638	0.063*	
C10	0.25731 (12)	0.40370 (17)	0.4193 (2)	0.0543 (7)	
H10	0.2474	0.3736	0.4803	0.065*	
C11	0.31271 (10)	0.41112 (16)	0.40178 (19)	0.0455 (6)	
C12	0.32510 (9)	0.45586 (14)	0.30937 (18)	0.0388 (5)	
H12	0.3619	0.4618	0.2952	0.047*	
C13	0.18268 (10)	0.52347 (19)	0.1809 (2)	0.0566 (7)	
H13A	0.1923	0.5194	0.1078	0.068*	
H13B	0.1770	0.5853	0.1990	0.068*	
H13C	0.1494	0.4902	0.1883	0.068*	
C14	0.35722 (12)	0.3700 (2)	0.4776 (2)	0.0622 (7)	
H14A	0.3417	0.3227	0.5179	0.075*	
H14B	0.3727	0.4151	0.5263	0.075*	
H14C	0.3856	0.3459	0.4369	0.075*	
N1	0.29977 (8)	0.52955 (13)	0.13736 (15)	0.0383 (4)	
H1N	0.3196 (9)	0.4971 (15)	0.1025 (18)	0.046*	
O1	0.26641 (6)	0.68499 (11)	0.15806 (13)	0.0464 (4)	
O2	0.32113 (7)	0.64305 (11)	0.00710 (12)	0.0478 (4)	
Cl1	0.43532 (3)	0.56301 (5)	0.07956 (5)	0.05525 (17)	
Cl2	0.51978 (3)	0.77011 (6)	0.40399 (7)	0.0827 (3)	
S1	0.31057 (2)	0.63530 (4)	0.11853 (4)	0.03675 (13)	
C15	0.10849 (8)	0.60065 (14)	0.88706 (17)	0.0341 (5)	
C16	0.10887 (9)	0.65759 (15)	0.97592 (17)	0.0371 (5)	
C17	0.14506 (9)	0.72894 (16)	0.98750 (18)	0.0417 (5)	
H17	0.1463	0.7652	1.0485	0.050*	
C18	0.17939 (9)	0.74567 (16)	0.90733 (19)	0.0417 (5)	
C19	0.17843 (10)	0.69315 (16)	0.8165 (2)	0.0456 (6)	
H19	0.2009	0.7067	0.7619	0.055*	
C20	0.14345 (9)	0.61981 (16)	0.80740 (18)	0.0415 (5)	
H20	0.1433	0.5828	0.7471	0.050*	
C21	−0.02186 (8)	0.57033 (16)	0.74447 (17)	0.0374 (5)	
C22	−0.02921 (10)	0.66073 (17)	0.7207 (2)	0.0472 (6)	
C23	−0.05413 (12)	0.6814 (2)	0.6184 (2)	0.0639 (8)	
H23	−0.0595	0.7413	0.5991	0.077*	
C24	−0.07089 (11)	0.6157 (2)	0.5453 (2)	0.0633 (8)	
H24	−0.0874	0.6322	0.4780	0.076*	
C25	−0.06385 (10)	0.5257 (2)	0.5695 (2)	0.0517 (6)	
C26	−0.03908 (9)	0.50430 (17)	0.67050 (19)	0.0436 (5)	
H26	−0.0338	0.4442	0.6892	0.052*	
C27	−0.01135 (13)	0.73384 (19)	0.7994 (3)	0.0681 (8)	
H27A	0.0279	0.7414	0.8015	0.082*	
H27B	−0.0213	0.7178	0.8700	0.082*	
H27C	−0.0293	0.7890	0.7771	0.082*	
C28	−0.08357 (13)	0.4536 (2)	0.4908 (2)	0.0771 (10)	
H28A	−0.0794	0.4737	0.4186	0.092*	
H28B	−0.1218	0.4410	0.4983	0.092*	
H28C	−0.0621	0.4001	0.5051	0.092*	
N2	0.00272 (8)	0.54195 (14)	0.84816 (15)	0.0397 (4)	
H2N	−0.0133 (10)	0.5490 (16)	0.9025 (16)	0.048*	
O3	0.07967 (7)	0.46077 (11)	0.77479 (13)	0.0462 (4)	
O4	0.06783 (7)	0.45703 (11)	0.96970 (13)	0.0476 (4)	
Cl3	0.06310 (3)	0.64443 (5)	1.07415 (5)	0.05622 (18)	
Cl4	0.22360 (3)	0.83646 (5)	0.92104 (6)	0.0652 (2)	
S2	0.06476 (2)	0.50518 (4)	0.86965 (4)	0.03674 (13)	
C29	0.29694 (9)	0.33765 (14)	0.81101 (17)	0.0355 (5)	
C30	0.24190 (9)	0.35952 (15)	0.82580 (18)	0.0397 (5)	
C31	0.19879 (10)	0.32037 (16)	0.7636 (2)	0.0472 (6)	
H31	0.1624	0.3349	0.7741	0.057*	
C32	0.21059 (11)	0.25911 (16)	0.6851 (2)	0.0479 (6)	
C33	0.26383 (11)	0.23590 (16)	0.66867 (19)	0.0466 (6)	
H33	0.2709	0.1943	0.6158	0.056*	
C34	0.30681 (10)	0.27513 (15)	0.73174 (18)	0.0411 (5)	
H34	0.3430	0.2595	0.7211	0.049*	
C35	0.37412 (9)	0.52663 (14)	0.75746 (17)	0.0367 (5)	
C36	0.33740 (10)	0.56036 (16)	0.67582 (19)	0.0444 (6)	
C37	0.35960 (11)	0.59760 (19)	0.5871 (2)	0.0562 (7)	
H37	0.3361	0.6217	0.5316	0.067*	
C38	0.41565 (12)	0.59976 (19)	0.5793 (2)	0.0609 (7)	
H38	0.4292	0.6246	0.5182	0.073*	
C39	0.45216 (11)	0.56595 (18)	0.6600 (2)	0.0541 (7)	
C40	0.43062 (10)	0.52978 (16)	0.7496 (2)	0.0451 (6)	
H40	0.4544	0.5072	0.8057	0.054*	
C41	0.27599 (11)	0.5588 (2)	0.6826 (2)	0.0670 (8)	
H41A	0.2679	0.5720	0.7551	0.080*	
H41B	0.2619	0.5004	0.6625	0.080*	
H41C	0.2589	0.6030	0.6344	0.080*	
C42	0.51378 (13)	0.5707 (3)	0.6520 (3)	0.0872 (11)	
H42A	0.5227	0.6260	0.6179	0.105*	
H42B	0.5252	0.5210	0.6101	0.105*	
H42C	0.5326	0.5682	0.7231	0.105*	
N3	0.35468 (8)	0.49250 (12)	0.85565 (15)	0.0384 (4)	
H3N	0.3345 (9)	0.5232 (15)	0.8909 (18)	0.046*	
O5	0.34473 (7)	0.38367 (11)	0.99890 (12)	0.0468 (4)	
O6	0.40211 (6)	0.34595 (11)	0.85093 (14)	0.0477 (4)	
Cl5	0.22596 (3)	0.43853 (5)	0.92023 (5)	0.05357 (17)	
Cl6	0.15616 (3)	0.21002 (6)	0.60671 (7)	0.0780 (2)	
S3	0.35411 (2)	0.38736 (4)	0.88676 (4)	0.03727 (13)	

### Atomic displacement parameters (Å^2^)

**Table:**

	*U*^11^	*U*^22^	*U*^33^	*U*^12^	*U*^13^	*U*^23^
C1	0.0373 (11)	0.0319 (11)	0.0368 (11)	0.0014 (9)	0.0061 (9)	0.0055 (9)
C2	0.0406 (12)	0.0384 (12)	0.0393 (12)	0.0058 (10)	0.0057 (10)	0.0018 (10)
C3	0.0383 (13)	0.0463 (14)	0.0589 (15)	0.0034 (11)	0.0053 (11)	−0.0005 (12)
C4	0.0510 (14)	0.0435 (14)	0.0504 (15)	−0.0018 (11)	−0.0053 (11)	0.0013 (11)
C5	0.0599 (16)	0.0412 (14)	0.0431 (13)	0.0054 (12)	0.0040 (12)	−0.0045 (11)
C6	0.0455 (13)	0.0387 (13)	0.0449 (13)	0.0058 (10)	0.0094 (11)	0.0019 (10)
C7	0.0384 (12)	0.0294 (11)	0.0373 (12)	−0.0009 (9)	0.0090 (9)	−0.0030 (9)
C8	0.0375 (12)	0.0390 (13)	0.0517 (14)	−0.0008 (10)	0.0105 (10)	−0.0070 (11)
C9	0.0457 (14)	0.0483 (15)	0.0669 (17)	−0.0044 (12)	0.0231 (13)	−0.0014 (13)
C10	0.0674 (17)	0.0469 (15)	0.0526 (15)	−0.0055 (13)	0.0272 (13)	0.0055 (12)
C11	0.0577 (15)	0.0362 (13)	0.0436 (13)	−0.0019 (11)	0.0096 (11)	0.0021 (10)
C12	0.0380 (12)	0.0347 (12)	0.0445 (13)	−0.0028 (9)	0.0078 (10)	−0.0004 (10)
C13	0.0374 (13)	0.0649 (18)	0.0675 (18)	0.0017 (12)	0.0040 (12)	−0.0047 (14)
C14	0.0745 (19)	0.0589 (17)	0.0530 (16)	0.0013 (15)	0.0048 (14)	0.0155 (13)
N1	0.0403 (11)	0.0387 (11)	0.0368 (10)	0.0018 (8)	0.0093 (8)	−0.0011 (8)
O1	0.0395 (9)	0.0445 (9)	0.0558 (10)	0.0102 (7)	0.0072 (7)	0.0018 (8)
O2	0.0497 (10)	0.0573 (11)	0.0365 (9)	0.0044 (8)	0.0047 (7)	0.0112 (8)
Cl1	0.0479 (3)	0.0647 (4)	0.0534 (4)	0.0141 (3)	0.0061 (3)	−0.0154 (3)
Cl2	0.0625 (5)	0.0876 (6)	0.0929 (6)	−0.0034 (4)	−0.0223 (4)	−0.0240 (5)
S1	0.0353 (3)	0.0389 (3)	0.0364 (3)	0.0050 (2)	0.0052 (2)	0.0058 (2)
C15	0.0293 (10)	0.0362 (11)	0.0366 (11)	0.0000 (9)	0.0017 (9)	0.0024 (9)
C16	0.0320 (11)	0.0454 (13)	0.0341 (11)	0.0007 (9)	0.0045 (9)	0.0022 (10)
C17	0.0452 (13)	0.0412 (13)	0.0381 (12)	−0.0032 (10)	−0.0004 (10)	−0.0029 (10)
C18	0.0385 (12)	0.0392 (13)	0.0468 (13)	−0.0072 (10)	0.0005 (10)	0.0056 (10)
C19	0.0427 (13)	0.0490 (14)	0.0466 (14)	−0.0050 (11)	0.0136 (11)	0.0029 (11)
C20	0.0430 (13)	0.0435 (13)	0.0391 (12)	−0.0021 (10)	0.0094 (10)	−0.0029 (10)
C21	0.0285 (10)	0.0463 (13)	0.0379 (12)	−0.0026 (9)	0.0055 (9)	0.0074 (10)
C22	0.0390 (13)	0.0453 (14)	0.0571 (15)	−0.0008 (10)	0.0029 (11)	0.0069 (12)
C23	0.0589 (17)	0.0561 (17)	0.075 (2)	0.0013 (14)	−0.0031 (15)	0.0264 (15)
C24	0.0519 (16)	0.085 (2)	0.0505 (16)	−0.0086 (15)	−0.0092 (13)	0.0214 (16)
C25	0.0401 (13)	0.0713 (19)	0.0438 (14)	−0.0142 (12)	0.0047 (11)	0.0035 (13)
C26	0.0387 (12)	0.0470 (14)	0.0455 (13)	−0.0083 (10)	0.0059 (10)	0.0050 (11)
C27	0.0690 (19)	0.0429 (15)	0.091 (2)	0.0041 (14)	−0.0038 (17)	−0.0007 (15)
C28	0.068 (2)	0.105 (3)	0.0572 (18)	−0.0259 (18)	−0.0007 (15)	−0.0113 (18)
N2	0.0341 (10)	0.0495 (12)	0.0359 (10)	−0.0009 (9)	0.0057 (8)	0.0063 (9)
O3	0.0443 (9)	0.0413 (9)	0.0532 (10)	−0.0003 (7)	0.0058 (7)	−0.0080 (8)
O4	0.0459 (9)	0.0474 (10)	0.0491 (10)	−0.0008 (8)	0.0023 (7)	0.0144 (8)
Cl3	0.0553 (4)	0.0695 (4)	0.0468 (3)	−0.0092 (3)	0.0211 (3)	−0.0064 (3)
Cl4	0.0691 (4)	0.0592 (4)	0.0670 (4)	−0.0302 (4)	0.0048 (3)	−0.0005 (3)
S2	0.0340 (3)	0.0359 (3)	0.0404 (3)	−0.0019 (2)	0.0032 (2)	0.0029 (2)
C29	0.0373 (11)	0.0322 (11)	0.0381 (12)	0.0039 (9)	0.0098 (9)	0.0061 (9)
C30	0.0427 (12)	0.0371 (12)	0.0407 (12)	0.0058 (10)	0.0120 (10)	0.0015 (10)
C31	0.0406 (13)	0.0447 (14)	0.0570 (15)	0.0050 (11)	0.0074 (11)	0.0031 (12)
C32	0.0526 (15)	0.0384 (13)	0.0514 (15)	0.0026 (11)	−0.0039 (12)	0.0006 (11)
C33	0.0602 (16)	0.0365 (13)	0.0435 (13)	0.0069 (11)	0.0075 (12)	−0.0030 (10)
C34	0.0449 (13)	0.0376 (12)	0.0422 (13)	0.0074 (10)	0.0116 (10)	0.0059 (10)
C35	0.0451 (13)	0.0286 (11)	0.0373 (12)	0.0003 (9)	0.0097 (10)	0.0000 (9)
C36	0.0485 (14)	0.0407 (13)	0.0446 (13)	0.0073 (11)	0.0080 (11)	0.0051 (11)
C37	0.0623 (17)	0.0594 (17)	0.0473 (15)	0.0129 (13)	0.0077 (13)	0.0170 (13)
C38	0.0698 (19)	0.0608 (18)	0.0554 (16)	0.0054 (15)	0.0241 (14)	0.0214 (14)
C39	0.0513 (15)	0.0510 (15)	0.0627 (17)	0.0016 (12)	0.0204 (13)	0.0103 (13)
C40	0.0416 (13)	0.0430 (13)	0.0511 (14)	0.0017 (10)	0.0063 (11)	0.0068 (11)
C41	0.0513 (16)	0.085 (2)	0.0652 (18)	0.0148 (15)	0.0054 (14)	0.0245 (16)
C42	0.0575 (19)	0.103 (3)	0.105 (3)	0.0012 (18)	0.0330 (18)	0.029 (2)
N3	0.0469 (11)	0.0332 (10)	0.0367 (10)	0.0046 (8)	0.0128 (8)	0.0012 (8)
O5	0.0526 (10)	0.0508 (10)	0.0372 (9)	0.0010 (8)	0.0051 (7)	0.0088 (7)
O6	0.0405 (9)	0.0440 (10)	0.0598 (10)	0.0093 (7)	0.0109 (8)	0.0056 (8)
Cl5	0.0490 (3)	0.0585 (4)	0.0551 (4)	0.0110 (3)	0.0150 (3)	−0.0127 (3)
Cl6	0.0680 (5)	0.0682 (5)	0.0927 (6)	0.0053 (4)	−0.0235 (4)	−0.0205 (4)
S3	0.0379 (3)	0.0366 (3)	0.0380 (3)	0.0047 (2)	0.0075 (2)	0.0061 (2)

### Geometric parameters (Å, °)

**Table:**

C1—C6	1.390 (3)	C23—C24	1.372 (4)
C1—C2	1.399 (3)	C23—H23	0.9300
C1—S1	1.780 (2)	C24—C25	1.381 (4)
C2—C3	1.381 (3)	C24—H24	0.9300
C2—Cl1	1.732 (2)	C25—C26	1.382 (3)
C3—C4	1.382 (3)	C25—C28	1.503 (4)
C3—H3	0.9300	C26—H26	0.9300
C4—C5	1.372 (4)	C27—H27A	0.9600
C4—Cl2	1.736 (3)	C27—H27B	0.9600
C5—C6	1.381 (3)	C27—H27C	0.9600
C5—H5	0.9300	C28—H28A	0.9600
C6—H6	0.9300	C28—H28B	0.9600
C7—C12	1.384 (3)	C28—H28C	0.9600
C7—C8	1.398 (3)	N2—S2	1.6049 (19)
C7—N1	1.442 (3)	N2—H2N	0.815 (16)
C8—C9	1.393 (3)	O3—S2	1.4261 (16)
C8—C13	1.502 (3)	O4—S2	1.4324 (16)
C9—C10	1.367 (4)	C29—C34	1.392 (3)
C9—H9	0.9300	C29—C30	1.404 (3)
C10—C11	1.387 (4)	C29—S3	1.772 (2)
C10—H10	0.9300	C30—C31	1.378 (3)
C11—C12	1.384 (3)	C30—Cl5	1.729 (2)
C11—C14	1.503 (4)	C31—C32	1.383 (3)
C12—H12	0.9300	C31—H31	0.9300
C13—H13A	0.9600	C32—C33	1.372 (3)
C13—H13B	0.9600	C32—Cl6	1.736 (3)
C13—H13C	0.9600	C33—C34	1.381 (3)
C14—H14A	0.9600	C33—H33	0.9300
C14—H14B	0.9600	C34—H34	0.9300
C14—H14C	0.9600	C35—C40	1.386 (3)
N1—S1	1.616 (2)	C35—C36	1.387 (3)
N1—H1N	0.830 (16)	C35—N3	1.439 (3)
O1—S1	1.4254 (16)	C36—C37	1.385 (3)
O2—S1	1.4356 (16)	C36—C41	1.502 (4)
C15—C20	1.389 (3)	C37—C38	1.374 (4)
C15—C16	1.392 (3)	C37—H37	0.9300
C15—S2	1.777 (2)	C38—C39	1.376 (4)
C16—C17	1.379 (3)	C38—H38	0.9300
C16—Cl3	1.733 (2)	C39—C40	1.381 (3)
C17—C18	1.377 (3)	C39—C42	1.511 (4)
C17—H17	0.9300	C40—H40	0.9300
C18—C19	1.373 (3)	C41—H41A	0.9600
C18—Cl4	1.726 (2)	C41—H41B	0.9600
C19—C20	1.382 (3)	C41—H41C	0.9600
C19—H19	0.9300	C42—H42A	0.9600
C20—H20	0.9300	C42—H42B	0.9600
C21—C26	1.386 (3)	C42—H42C	0.9600
C21—C22	1.386 (3)	N3—S3	1.6128 (19)
C21—N2	1.437 (3)	N3—H3N	0.824 (16)
C22—C23	1.396 (4)	O5—S3	1.4335 (16)
C22—C27	1.503 (4)	O6—S3	1.4243 (16)
			
C6—C1—C2	118.6 (2)	C25—C24—H24	119.2
C6—C1—S1	117.97 (17)	C24—C25—C26	117.2 (2)
C2—C1—S1	123.44 (17)	C24—C25—C28	121.6 (3)
C3—C2—C1	120.7 (2)	C26—C25—C28	121.2 (3)
C3—C2—Cl1	117.63 (18)	C25—C26—C21	121.5 (2)
C1—C2—Cl1	121.66 (18)	C25—C26—H26	119.2
C2—C3—C4	118.9 (2)	C21—C26—H26	119.2
C2—C3—H3	120.6	C22—C27—H27A	109.5
C4—C3—H3	120.6	C22—C27—H27B	109.5
C5—C4—C3	121.7 (2)	H27A—C27—H27B	109.5
C5—C4—Cl2	119.3 (2)	C22—C27—H27C	109.5
C3—C4—Cl2	119.0 (2)	H27A—C27—H27C	109.5
C4—C5—C6	119.0 (2)	H27B—C27—H27C	109.5
C4—C5—H5	120.5	C25—C28—H28A	109.5
C6—C5—H5	120.5	C25—C28—H28B	109.5
C5—C6—C1	121.0 (2)	H28A—C28—H28B	109.5
C5—C6—H6	119.5	C25—C28—H28C	109.5
C1—C6—H6	119.5	H28A—C28—H28C	109.5
C12—C7—C8	121.1 (2)	H28B—C28—H28C	109.5
C12—C7—N1	119.03 (19)	C21—N2—S2	124.18 (15)
C8—C7—N1	119.6 (2)	C21—N2—H2N	120.9 (18)
C9—C8—C7	116.1 (2)	S2—N2—H2N	114.6 (18)
C9—C8—C13	120.4 (2)	O3—S2—O4	119.25 (10)
C7—C8—C13	123.5 (2)	O3—S2—N2	108.80 (10)
C10—C9—C8	122.5 (2)	O4—S2—N2	107.06 (10)
C10—C9—H9	118.7	O3—S2—C15	106.25 (10)
C8—C9—H9	118.7	O4—S2—C15	107.97 (10)
C9—C10—C11	121.4 (2)	N2—S2—C15	106.93 (10)
C9—C10—H10	119.3	C34—C29—C30	118.2 (2)
C11—C10—H10	119.3	C34—C29—S3	118.74 (17)
C12—C11—C10	116.9 (2)	C30—C29—S3	123.09 (17)
C12—C11—C14	121.4 (2)	C31—C30—C29	121.0 (2)
C10—C11—C14	121.7 (2)	C31—C30—Cl5	117.74 (18)
C11—C12—C7	122.0 (2)	C29—C30—Cl5	121.21 (18)
C11—C12—H12	119.0	C30—C31—C32	118.8 (2)
C7—C12—H12	119.0	C30—C31—H31	120.6
C8—C13—H13A	109.5	C32—C31—H31	120.6
C8—C13—H13B	109.5	C33—C32—C31	121.8 (2)
H13A—C13—H13B	109.5	C33—C32—Cl6	119.6 (2)
C8—C13—H13C	109.5	C31—C32—Cl6	118.7 (2)
H13A—C13—H13C	109.5	C32—C33—C34	119.1 (2)
H13B—C13—H13C	109.5	C32—C33—H33	120.5
C11—C14—H14A	109.5	C34—C33—H33	120.5
C11—C14—H14B	109.5	C33—C34—C29	121.2 (2)
H14A—C14—H14B	109.5	C33—C34—H34	119.4
C11—C14—H14C	109.5	C29—C34—H34	119.4
H14A—C14—H14C	109.5	C40—C35—C36	121.0 (2)
H14B—C14—H14C	109.5	C40—C35—N3	118.1 (2)
C7—N1—S1	124.04 (15)	C36—C35—N3	120.8 (2)
C7—N1—H1N	115.5 (17)	C37—C36—C35	117.2 (2)
S1—N1—H1N	112.4 (18)	C37—C36—C41	120.5 (2)
O1—S1—O2	119.58 (10)	C35—C36—C41	122.2 (2)
O1—S1—N1	108.61 (10)	C38—C37—C36	121.5 (2)
O2—S1—N1	105.24 (10)	C38—C37—H37	119.3
O1—S1—C1	105.90 (10)	C36—C37—H37	119.3
O2—S1—C1	108.53 (10)	C37—C38—C39	121.4 (2)
N1—S1—C1	108.64 (10)	C37—C38—H38	119.3
C20—C15—C16	118.6 (2)	C39—C38—H38	119.3
C20—C15—S2	118.27 (17)	C38—C39—C40	117.8 (2)
C16—C15—S2	123.16 (16)	C38—C39—C42	121.0 (3)
C17—C16—C15	120.8 (2)	C40—C39—C42	121.2 (3)
C17—C16—Cl3	117.13 (17)	C39—C40—C35	121.1 (2)
C15—C16—Cl3	122.00 (17)	C39—C40—H40	119.5
C18—C17—C16	119.0 (2)	C35—C40—H40	119.5
C18—C17—H17	120.5	C36—C41—H41A	109.5
C16—C17—H17	120.5	C36—C41—H41B	109.5
C19—C18—C17	121.6 (2)	H41A—C41—H41B	109.5
C19—C18—Cl4	119.60 (18)	C36—C41—H41C	109.5
C17—C18—Cl4	118.75 (19)	H41A—C41—H41C	109.5
C18—C19—C20	118.9 (2)	H41B—C41—H41C	109.5
C18—C19—H19	120.5	C39—C42—H42A	109.5
C20—C19—H19	120.5	C39—C42—H42B	109.5
C19—C20—C15	120.9 (2)	H42A—C42—H42B	109.5
C19—C20—H20	119.5	C39—C42—H42C	109.5
C15—C20—H20	119.5	H42A—C42—H42C	109.5
C26—C21—C22	121.4 (2)	H42B—C42—H42C	109.5
C26—C21—N2	117.7 (2)	C35—N3—S3	123.80 (15)
C22—C21—N2	120.8 (2)	C35—N3—H3N	120.6 (18)
C21—C22—C23	116.5 (2)	S3—N3—H3N	113.2 (18)
C21—C22—C27	122.7 (2)	O6—S3—O5	119.40 (10)
C23—C22—C27	120.9 (2)	O6—S3—N3	108.72 (10)
C24—C23—C22	121.8 (3)	O5—S3—N3	106.01 (10)
C24—C23—H23	119.1	O6—S3—C29	106.19 (10)
C22—C23—H23	119.1	O5—S3—C29	108.55 (10)
C23—C24—C25	121.5 (3)	N3—S3—C29	107.47 (10)
C23—C24—H24	119.2		
			
C6—C1—C2—C3	−2.8 (3)	C22—C23—C24—C25	−0.1 (4)
S1—C1—C2—C3	175.64 (18)	C23—C24—C25—C26	−0.1 (4)
C6—C1—C2—Cl1	175.86 (17)	C23—C24—C25—C28	178.3 (3)
S1—C1—C2—Cl1	−5.7 (3)	C24—C25—C26—C21	−0.1 (4)
C1—C2—C3—C4	2.1 (4)	C28—C25—C26—C21	−178.6 (2)
Cl1—C2—C3—C4	−176.62 (19)	C22—C21—C26—C25	0.6 (3)
C2—C3—C4—C5	−0.1 (4)	N2—C21—C26—C25	179.0 (2)
C2—C3—C4—Cl2	179.23 (19)	C26—C21—N2—S2	76.8 (2)
C3—C4—C5—C6	−1.1 (4)	C22—C21—N2—S2	−104.7 (2)
Cl2—C4—C5—C6	179.57 (19)	C21—N2—S2—O3	−31.0 (2)
C4—C5—C6—C1	0.3 (4)	C21—N2—S2—O4	−161.10 (18)
C2—C1—C6—C5	1.6 (3)	C21—N2—S2—C15	83.4 (2)
S1—C1—C6—C5	−176.95 (18)	C20—C15—S2—O3	5.4 (2)
C12—C7—C8—C9	−0.5 (3)	C16—C15—S2—O3	−175.35 (18)
N1—C7—C8—C9	173.5 (2)	C20—C15—S2—O4	134.45 (18)
C12—C7—C8—C13	179.6 (2)	C16—C15—S2—O4	−46.3 (2)
N1—C7—C8—C13	−6.4 (3)	C20—C15—S2—N2	−110.64 (18)
C7—C8—C9—C10	0.2 (4)	C16—C15—S2—N2	68.6 (2)
C13—C8—C9—C10	−180.0 (2)	C34—C29—C30—C31	−0.1 (3)
C8—C9—C10—C11	0.4 (4)	S3—C29—C30—C31	178.74 (18)
C9—C10—C11—C12	−0.6 (4)	C34—C29—C30—Cl5	−178.42 (17)
C9—C10—C11—C14	−178.7 (2)	S3—C29—C30—Cl5	0.4 (3)
C10—C11—C12—C7	0.2 (3)	C29—C30—C31—C32	−0.4 (4)
C14—C11—C12—C7	178.3 (2)	Cl5—C30—C31—C32	177.96 (18)
C8—C7—C12—C11	0.4 (3)	C30—C31—C32—C33	0.6 (4)
N1—C7—C12—C11	−173.7 (2)	C30—C31—C32—Cl6	−179.90 (18)
C12—C7—N1—S1	−96.7 (2)	C31—C32—C33—C34	−0.3 (4)
C8—C7—N1—S1	89.1 (2)	Cl6—C32—C33—C34	−179.79 (18)
C7—N1—S1—O1	−47.2 (2)	C32—C33—C34—C29	−0.2 (3)
C7—N1—S1—O2	−176.39 (17)	C30—C29—C34—C33	0.4 (3)
C7—N1—S1—C1	67.5 (2)	S3—C29—C34—C33	−178.48 (18)
C6—C1—S1—O1	4.7 (2)	C40—C35—C36—C37	−0.4 (3)
C2—C1—S1—O1	−173.71 (18)	N3—C35—C36—C37	175.7 (2)
C6—C1—S1—O2	134.30 (17)	C40—C35—C36—C41	−179.3 (2)
C2—C1—S1—O2	−44.2 (2)	N3—C35—C36—C41	−3.2 (4)
C6—C1—S1—N1	−111.77 (18)	C35—C36—C37—C38	1.1 (4)
C2—C1—S1—N1	69.8 (2)	C41—C36—C37—C38	180.0 (3)
C20—C15—C16—C17	−3.2 (3)	C36—C37—C38—C39	−0.8 (5)
S2—C15—C16—C17	177.61 (17)	C37—C38—C39—C40	−0.2 (4)
C20—C15—C16—Cl3	174.91 (17)	C37—C38—C39—C42	−178.4 (3)
S2—C15—C16—Cl3	−4.3 (3)	C38—C39—C40—C35	0.8 (4)
C15—C16—C17—C18	2.8 (3)	C42—C39—C40—C35	179.0 (3)
Cl3—C16—C17—C18	−175.37 (18)	C36—C35—C40—C39	−0.5 (4)
C16—C17—C18—C19	0.0 (4)	N3—C35—C40—C39	−176.7 (2)
C16—C17—C18—Cl4	178.69 (17)	C40—C35—N3—S3	−75.5 (3)
C17—C18—C19—C20	−2.3 (4)	C36—C35—N3—S3	108.3 (2)
Cl4—C18—C19—C20	178.97 (19)	C35—N3—S3—O6	37.0 (2)
C18—C19—C20—C15	1.9 (4)	C35—N3—S3—O5	166.52 (18)
C16—C15—C20—C19	0.8 (3)	C35—N3—S3—C29	−77.5 (2)
S2—C15—C20—C19	−179.97 (18)	C34—C29—S3—O6	−3.0 (2)
C26—C21—C22—C23	−0.8 (3)	C30—C29—S3—O6	178.14 (18)
N2—C21—C22—C23	−179.2 (2)	C34—C29—S3—O5	−132.55 (17)
C26—C21—C22—C27	179.6 (2)	C30—C29—S3—O5	48.6 (2)
N2—C21—C22—C27	1.2 (4)	C34—C29—S3—N3	113.20 (18)
C21—C22—C23—C24	0.5 (4)	C30—C29—S3—N3	−65.6 (2)
C27—C22—C23—C24	−179.8 (3)		

### Hydrogen-bond geometry (Å, °)

**Table:**

*D*—H···*A*	*D*—H	H···*A*	*D*···*A*	*D*—H···*A*
N1—H1N···O5^i^	0.83 (2)	2.24 (2)	3.033 (2)	160 (2)
N2—H2N···O4^ii^	0.82 (2)	2.16 (2)	2.956 (2)	167 (2)
N3—H3N···O2^iii^	0.82 (2)	2.34 (2)	3.082 (2)	151 (2)

Symmetry codes: (i) *x*, *y*, *z*−1; (ii) −*x*, −*y*+1, −*z*+2; (iii) *x*, *y*, *z*+1.
